# Author's response to Poole, C. Commentary: How Many Are Affected? A Real Limit of Epidemiology

**DOI:** 10.1186/1742-5573-7-7

**Published:** 2010-08-26

**Authors:** Nicolle M Gatto, Ulka B Campbell, Sharon Schwartz

**Affiliations:** 1 Department of Epidemiology, Mailman School of Public Health, Columbia University, New York, NY 10032 USA; 2 Epidemiology, Worldwide Safety Strategy, Pfizer Inc, New York, NY 10017 USA

## 

We read with interest Charlie Poole's commentary [1] on our paper, "Redundant causation from a sufficient cause perspective,"[2] in which he questions the utility of the sufficient component cause (SCC) model for examining differences between etiologic and excess effects. Poole contends that the concept we term "redundant causation" is uncomplicated and (we presume), well understood. He questions whether "it needs to be explained in terms any deeper than those of potential outcomes" [1]. His critique of our paper focuses on our hypothetical and simplistic example of sufficient causes (SCs) of liver cancer. To be of value, Poole believes our example must be realistic and must bring "aspects of the potential outcome and sufficient cause models, and their interface, into sharp relief" [1]. His concerns raise larger issues about the roles of simplifications and the SCC model in methods research in general. We address each of these below.

## Simplifications

As Poole suggests, the conceptual and mathematical underpinnings of redundant causation (which we refer to colloquially as "redundancy") and its potential impact on etiologic versus excess effects have been explained by others in philosophy and epidemiology [3-10]. We disagree, however, that redundancy is "uncomplicated", fully examined in the literature, or well understood by epidemiologists. One purpose of our paper was to make the discussion of redundancy more accessible to a broad epidemiologic audience. To this end, we used a hypothetical and intentionally simplified liver cancer example to help crystallize this discussion for those who prefer tangible examples.

Poole was bothered by many simplifications in our liver cancer example, including the lack of competing risks, lack of disease recurrence, lack of shared causal components and presence of ubiquitous components. Although these simplifications may be unrealistic, we feel they were appropriate for illustration purposes. Indeed, the use of simplifying assumptions is ubiquitous in methodological work. To discover underlying principles, the complexity of real life situations must be controlled. Charges of oversimplification, therefore, should be grounded in a discussion of which simplifying assumptions are legitimate and which are not.

It seems to us that simplifying assumptions are legitimate if they are: (1) possible, or (2) impossible (or highly unlikely), but violating the assumption does not change the argument. For example, a monotonicity assumption is often invoked in methods work; it is often assumed that a risk factor is either causal or protective for the disease, but not both. This assumption is legitimate because it is possible. There are risk factors that meet this assumption. One can validly illustrate a methods principle invoking this assumption because there are realistic circumstances under which this principle would hold. Once the principle is understood within this context, the next step would be to examine the impact of relaxing this assumption [11,12].

Another frequently invoked assumption is an infinitely large sample size (see for example, [13]). This assumption is, by definition, impossible - no study can have an infinitely large sample. Nonetheless, this assumption can be legitimate because it often does not change the principle under investigation; it just allows the principle to be explicated more clearly.

In our liver cancer example, we think that all the simplifications meet the criteria for legitimate assumptions. Our assumptions are either possible (e.g., that alcohol consumption is the final component to complete the sufficient cause), or impossible but do not change the argument (e.g., that no one dies during the study period).

Ironically, in the methods literature, the absence of redundancy is often invoked as a simplifying assumption. Although Poole suggests that redundancy is well understood, its inevitability is often disregarded in methods work. That is, "no redundancy" is an impossible assumption that sometimes does change the principles under study. This illegitimacy of the "no redundancy" assumption became apparent when the effects of parallelism on the assessment of synergy entered the literature [14] and is a problem, we think, in some of the literature on mediation [15,16].

By invoking simplifying assumptions in our liver cancer example, we showed that the presence of even one individual with redundant sufficient causes can lead to a discrepancy between an etiologic and excess effect measure. To us, the next natural questions are "under what circumstances will redundancy lead to large discrepancies between etiologic and excess effects?" followed by "how realistic are those circumstances"? We have begun to address these additional questions (in manuscripts that are in various states of preparation), and we hope that other epidemiologists will be intrigued and help round out the answers.

## Utility of the SCC model

Many methodologic innovations in epidemiology have been forged using POs. In recent years, drawing connections between POs and SCCs seems to have become a topic of interest [10,12,17-24]. However, many of these discussions start with a set of POs and then connect them to an underlying general SCC model. We (the authors) begin methodologic research by specifying the directed acyclic graph (DAG) that depicts the relationships we intend to examine. From there, we apply the DAG rules to draw a SCC model that underlies the DAG. The potential outcomes arise directly from that specific SCC model and the prevalences of its components. We recognize that our perspective on the links among a DAG, SSC model, and set of POs is not typical; we have begun to address this elsewhere [25,26].

The SCC model has been used to illustrate several epidemiologic principles in the literature. For instance, in the Modern Epidemiology text, Rothman and Greenland use a SCC model to show how the strength of an association is caused by the prevalence of the causal partner [27] and to demonstrate the concepts of synergy and parallelism -- that the extent of synergy is determined by the prevalences of all partners of the synergistic factors, and that additive interaction is synergy minus parallelism [28]. Notably, the most recent version of Modern Epidemiology invokes a simple SCC model to describe how redundant causation arises [29].

The second purpose of our paper was to make the discussion of redundancy more comprehensive. Like parallelism, redundancy is most clearly seen through the SCC lens. The SCC model makes clear: 1) how redundancy arises, 2) that it is a naturally occurring, *inevitable *phenomenon, and 3) which factors influence the proportion of redundant individuals in a population for a particular disease at a particular point in time. POs allow us to see the critical mathematical distinction between disease etiology and excess, but do not allow us to see what factors influence the distribution of the POs, which ultimately drives the strength of the etiologic effect and thus its discrepancy from the excess effect we actually measure.

## Conclusions

Our examination of redundancy shows that even in the simplest (and perhaps most unrealistic) scenarios (e.g. when none of the sufficient causes shares components and there are no associations between components), redundancy can influence our effect estimates. It appears that the more complicated the SCC model (e.g., the more SCs, shared components, or associations between components in the SCC model), the more redundant individuals there will be, and the larger the discrepancy between the etiologic and excess effects. We do not believe that these principles could be revealed using potential outcomes alone.

## References

[B1] Poole C Commentary: How Many Are Affected? A Real Limit of Epidemiology Epidemiol Perspect Innov 2010 7 1 6 20735844 10.1186/1742-5573-7-6 PMC2936291

[B2] Gatto NM Campbell UB Redundant causation from a sufficient cause perspective Epidemiol Perspect Innov 2010 7 5 20678223 10.1186/1742-5573-7-5 PMC2922199

[B3] Beyea J Greenland S The importance of specifying the underlying biologic model in estimating the probability of causation Health Phys 1999 76 269 74 10.1097/00004032-199903000-00008 10025652

[B4] Greenland S Relation of probability of causation to relative risk and doubling dose: a methodologic error that has become a social problem Am J Public Health 1999 89 1166 9 10.2105/AJPH.89.8.1166 10432900 PMC1508676

[B5] Greenland S Robins JM Conceptual problems in the definition and interpretation of attributable fractions Am J Epidemiol 1988 128 1185 97 3057878 10.1093/oxfordjournals.aje.a115073

[B6] Mackie JL The Cement of the Universe: A Study of Causation 1974 New York, NY: Oxford University Press

[B7] Robins J Greenland S The probability of causation under a stochastic model for individual risk Biometrics 1989 45 1125 38 10.2307/2531765 2611320

[B8] Robins JM Greenland S Estimability and estimation of excess and etiologic fractions Stat Med 1989 8 845 59 10.1002/sim.4780080709 2772444

[B9] Rothman KJ Causes Am J Epidemiol 1976 104 587 92 998606 10.1093/oxfordjournals.aje.a112335

[B10] Rothman KJ Greenland S Lash TL Modern Epidemiology 2008 Philadelphia, PA: Lippincott Williams & Wilkins

[B11] VanderWeele TJ Attributable Fractions for Sufficient Cause Interactions The International Journal of Biostatistics 2010 5 1 26 10.2202/1557-4679.1202 PMC2836214 20305707

[B12] VanderWeele TJ Robins JM Empirical and counterfactual conditions for sufficient cause interactions Biometrika 2008 95 49 61 10.1093/biomet/asm090

[B13] Hernan MA Robins JM Estimating causal effects from epidemiological data J Epidemiol Community Health 2006 60 578 86 10.1136/jech.2004.029496 16790829 PMC2652882

[B14] Darroch J Biologic synergism and parallelism Am J Epidemiol 1997 145 661 8 9098184 10.1093/oxfordjournals.aje.a009164

[B15] Kaufman S Kaufman JS Maclehose RF Improved estimation of controlled direct effects in the presence of unmeasured confounding of intermediate variables Stat Med 2005 24 1683 702 10.1002/sim.2057 15742358

[B16] Robins JM Greenland S Identifiability and exchangeability for direct and indirect effects Epidemiology 1992 3 143 55 10.1097/00001648-199203000-00013 1576220

[B17] Flanders WD On the relationship of sufficient component cause models with potential outcome (counterfactual) models Eur J Epidemiol 2006 21 847 53 10.1007/s10654-006-9048-3 17048084

[B18] VanderWeele TJ Hernan MA From counterfactuals to sufficient component causes and vice versa Eur J Epidemiol 2007 21 855 8 10.1007/s10654-006-9075-0 17225959

[B19] Greenland S Gelman A, Meng X-L An overview of methods for causal inference from observational studies Applied Bayesian Modeling and Causal Inference from Incomplete-Data Perspectives 2004 West Sussex, England: John Wiley & Sons Ltd 3 13

[B20] Greenland S Brumback B An overview of relations among causal modelling methods Int J Epidemiol 2002 31 1030 7 10.1093/ije/31.5.1030 12435780

[B21] Greenland S Poole C Invariants and noninvariants in the concept of interdependent effects Scand J Work Environ Health 1988 14 125 9 3387960 10.5271/sjweh.1945

[B22] VanderWeele TJ Mediation and mechanism Eur J Epidemiol 2009 24 217 24 10.1007/s10654-009-9331-1 19330454

[B23] VanderWeele TJ Robins JM The identification of synergism in the sufficient-component cause framework Epidemiology 2007 18 329 39 10.1097/01.ede.0000260218.66432.88 17435441

[B24] VanderWeele TJ Robins JM Directed acyclic graphs, sufficient causes, and the properties of conditioning on a common effect Am J Epidemiol 2007 166 1096 104 10.1093/aje/kwm179 17702973

[B25] Gatto NM Campbell UB Hodge SE A simulation program for epidemiologists, by epidemiologists Presented at Society for Epidemiologic Research (SER) Annual Meeting, Toronto, Canada (June 2005)

[B26] Schwartz S Gatto NM Campbell UB Shrout P What would have been is not what would be: counterfactuals of the past and potential outcomes of the future Causality and Psychopathology: Finding the Determinants of Disorders and Their Cures New York: American Psychiatric Publishers Inc in press

[B27] Rothman KJ Greenland S Poole C Rothman KJ, Greenland S, Lash TL Causation and causal inference Modern Epidemiology 2008 Philadelphia, PA: Lippincott WIlliams & Wilkins 5 31

[B28] Greenland S Lash TL Rothman KJ Rothman KJ, Greenland S, Lash TL Concepts of Interaction Modern Epidemiology 2008 Philadelphia, PA: Lippincott WIlliams & Wilkins 71 86

[B29] Greenland S Rothman KJ Lash TL Rothman KJ, Greenland S, Lash TL Measures of Effect and Measures of Association Modern Epidemiology 2008 Philadelphia, PA: Lippincott WIlliams & Wilkins 51 70

